# Can online health information sources really improve patient satisfaction?

**DOI:** 10.3389/fpubh.2022.940800

**Published:** 2022-08-05

**Authors:** Yide Sun, Jiajun Yu, Ya-Ling Chiu, Yuan-Teng Hsu

**Affiliations:** ^1^College of International Business, Zhejiang Yuexiu University, Shaoxing, China; ^2^Shaoxing Key Laboratory for Smart Society Monitoring, Prevention and Control, Shaoxing, China; ^3^School of Innovation and Entrepreneurship, Guangzhou Huashang College, Guangzhou, China; ^4^School of Management, Guangzhou Huashang College, Guangzhou, China; ^5^Research Center of Finance, Shanghai Business School, Shanghai, China

**Keywords:** online information sources, patient satisfaction, decision-making, online healthcare community, internet information

## Abstract

Internet information has become the main way for individuals to obtain health information. The purpose of this paper is to explore the role online information sources play in health decision-making. Specifically, we investigated the relationship between online information sources and patient satisfaction, as well as their moderating effects as compared to those of other information sources. Using logistical regression analysis, we conducted the longitudinal data on 54,027 doctors and 952,877 online doctor reviews from 3,525 hospitals in 31 provinces to test a proposed research model. The results showed that patient satisfaction was generally lower for individuals who found a doctor through online information sources. Therefore, we suggest that patients consider the doctor quality, the doctor popularity, and patient involvement. In addition, we found that the doctor popularity had a negative moderating effect between online information sources and patient satisfaction, while patient involvement had a positive moderating effect between online information sources and patient satisfaction. The study provides strategic guidance and practical implications for policies, online healthcare community managers, and patients.

## Introduction

The Internet is changing the way information is delivered to consumers and the way consumers act on information. Due to the convenience, immediacy, and interactivity of the Internet, more and more consumers are turning to it to seek health information (1–5). In the United States, 72% of Internet users reported that they had looked for health information online in the preceding 12 months (6). According to the 2019 EU citizens' use ICT survey, 76% of Internet users in Finland searched for health-related topics online, followed by users in the Netherlands (74%), Cyprus (69%), Denmark (67%), and Germany (66%). In contrast, Bulgaria (30%), Romania (31%) and Italy (35%) had the lowest rates of use of the Internet to search for health-related topics (7). Furthermore, according to a survey conducted by the Kantar (8) and DXY^1^ in China, patients spent ~28 h per week online, of which 8 h (28.6%) were spent searching for medical information. Therefore, it is clear that online health information is becoming increasingly more important for patients.

Much of the previous research on online health information seeking has focused on understanding the people who seek health information online and how they use online health information (9, 10), but little is known about whether customers make better decisions when using online health information sources. Specifically, patients who wanted to find a doctor would traditionally obtain health information from healthcare providers, mass media, or local community members, i.e., members of groups who are considered to have certain health knowledge (11). In recent years, the Internet has provided patients with a convenient way to broadly understand their disease and its prevention methods (12, 13) and even how to choose the right doctor (14). In other words, online health information has become an important information source for medical decision-making (14, 15). The purpose of this study was to answer the following questions: (1) What is the impact of various sources of information on patient satisfaction? (2) How do the information source preferred by patients in choosing a doctor change when the quality of the doctor, the popularity of the doctor, and the level of patient involvement?

A recent study by Zhang et al. (14) pointed out that online patient reviews, family and friend recommendations, and doctor recommendations were three primary information sources that tended to be patients' preferred sources of information for choosing physicians based on their own circumstances. This study extends the results of Zhang et al. (14) to further discuss whether online health information sources can really improve customer health-related decision-making and what the moderating effects are in different situations. Specifically, the study explores the impact of whether patients choose doctors through the online or other information sources (i.e., family and friend sources or doctor sources) on patient satisfaction. This study uses patient satisfaction to evaluate the quality of decision-making; patient satisfaction includes treatment effect satisfaction and service attitude satisfaction (16). In addition, we use doctor quality, doctor popularity, and patient involvement as moderator variables. We provide empirical support to fill the gaps in the research by exploring the impact of online health information sources on patient satisfaction and further testing its moderating effects. To achieve the aforementioned objectives, we collected data on patient satisfaction and other relevant data from a the Haodf website (http://haodf.com) in China from September 2017 to August 2019, using empirical analysis to test our hypotheses.

The contributions of this study are threefold. First, although patient satisfaction has been an extensively researched topic recently, most previous studies on patient satisfaction have used a cross-sectional questionnaire and descriptive studies, which makes it difficult to establish a causal relationship between determinants and satisfaction. Thus, a longitudinal research design is needed to detect causal relationships (17, 18). To investigate whether online health information sources would cause patient satisfaction, we designed a longitudinal study. Data were collected once a month for two consecutive years, including information on 54,027 doctors and 952,877 online doctor reviews from 3,525 hospitals in 31 provinces. The comprehensive results facilitate a clearer understanding of the determinants of patient satisfaction. Second, following the theory of recommendation sources in consumer decision-making, many studies (19, 20) have shown that online consumer reviews are the primary source of information consumers use for their decisions. Previous research has focused on the impact of online consumer reviews on consumer choice, while few studies have examined customer satisfaction arising from the use of online information sources. To the best of our knowledge, this study is the first to use a large amount of online healthcare community (OHC) data to investigate the impact of online health information sources on patient satisfaction. We found an interesting result, that patients who found a doctor through online health information sources were generally less satisfied. We thus suggest that patients consider other moderator variables. Third, we found that a doctor's popularity strengthens the negative impact of online health information sources on patient satisfaction, while patient involvement weakens the negative association between online health information sources and patient satisfaction. However, the results on doctor quality do not support our proposed hypothesis. Our study included an in-depth analysis of online health information sources and patient satisfaction and made findings with the potential to enrich the application of recommendation sources theory in the medical field. Based on our findings, we provide practical management implications for patients who use online health information sources to select doctors. Furthermore, although online information is an important source for customer decision making, it has a negative impact on patient satisfaction in the healthcare context, a finding which points to managerial implications for OHC managers.

The rest of this paper is organized as follows. The next section presents a brief overview of prior research and the theoretical foundation of this study. In section Research methods, we describe our research methodology. We then present the data analysis and results in Section Results. Finally, we conclude with a discussion of implications, limitations, and future research.

## Related literature and hypotheses

**Figure 1:**
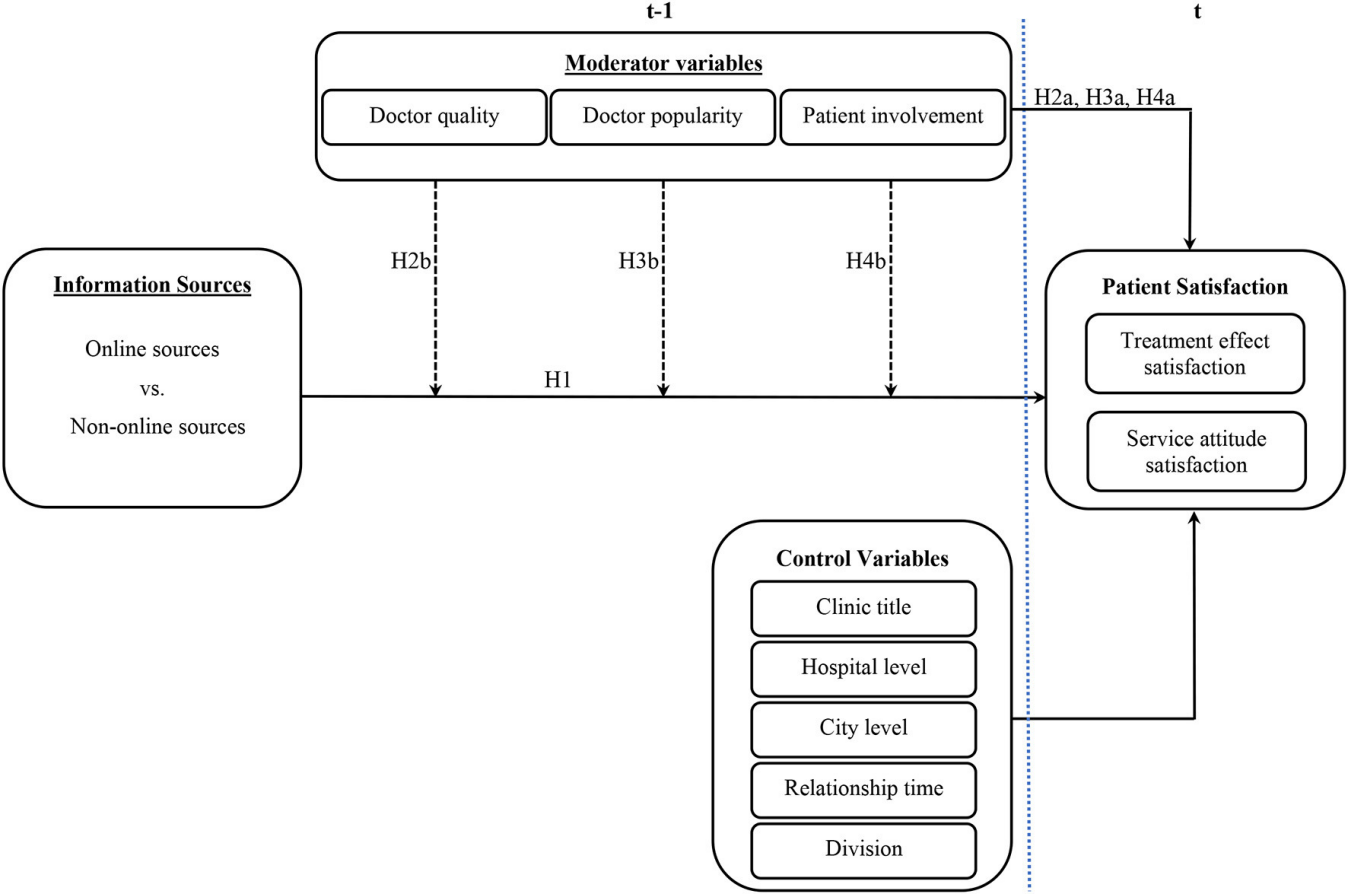
Research framework.

Based on the literature on health information sources, health-related decision-making and patient satisfaction, this study investigated the relationship between online health information sources and patient satisfaction and tested for the moderating effect of online health information sources on patient satisfaction. We propose the conceptual framework shown in Figure 1. Based on the relationship expressed in the framework of Figure 1, the following hypotheses were developed.

### Health information sources and health-related decision-making

Individuals may choose and use different information sources to make decisions. The choice of information sources may depend on factors such as quality and accessibility (21). Given that different health information sources have varying degrees of credibility, especially now that online information is becoming more prevalent, it is important to determine whether consumers are better off using online health information sources for decision making when finding a doctor. Existing studies provides some insights into users' preferences for sources of health information (14, 22, 23). For example, Couper et al. (22) found that adult Internet users and non-users aged 40 and older in the U.S. used physicians as their primary source of information when making medical decisions. The most influential source of information for Internet users was health care providers, followed by the Internet, family and friends, and the media. Hall et al. (23) found significant differences between older adult users and non-users of online and offline health information sources. Online health information users preferred the self-reliance approach, while non-users preferred the physician-dependent approach. A recent study by Zhang et al. (14) has shown that online patient reviews, recommendations from family and friends, and recommendations from physicians were the three main sources of information used in China. These information sources were significantly associated with patients' choice of preferred sources of information about their physicians based on their circumstances (involving for example the difficulty of medical decision-making, the level of hospitals, and urban/rural areas differences). With the rapid development of the Internet, online health information has become an important source for patients who are seeking information (1–5). While these studies shed light on patients' use of information sources when making medical decisions, findings are limited. Specifically, discussion of whether online health information sources can really improve customer decision-making and what the moderating effects are in different situations has been absent.

### Online health information sources and patient satisfaction

This study explores the relationship between different health information sources and decision-making outcomes. Previous studies have demonstrated that seeking health information online can increase patient knowledge and patients' ability to participate in decision-making, improve their understanding of medical health outcomes, and ultimately increase patient satisfaction (24). In this study, patient satisfaction was seen as the outcome of good or bad decision making. After receiving treatment and services from a physician, a patient will evaluate the effectiveness of the doctor's treatment and the doctor's service attitude. Patient satisfaction is an affective evaluation of the service based on cognitive processes, which are shaped by expectations. When the perceived quality of service exceeds expectations, patients are satisfied with physician services (25). Previous studies have shown that an assessment of patient satisfaction with health care services can improve the quality of health care by identifying problems (26). (27) indicated that patients rate the quality of healthcare in light of various aspects, including explanation and the effect of treatment, the decision-making process, and health care organization. We measured patient satisfaction in terms of both treatment effect satisfaction and service attitude satisfaction.

With the development of a mature OHC, many people can now use the review ratings and information available within the OHC to understand the service quality of their doctors. The theory of the Weak Ties Network explores the importance of interpersonal relationships in the process of information distribution (28, 29). According to this theory, online information provided by mere acquaintances or people who do not know the patient at all can be considered weak-tie information, while information sources such as friends or family numbers are strong-tie sources (30). The advantage of strong-tie sources is that people can evaluate the alternatives based on their individual circumstances. People are more likely to support those with whom they have strong ties than those with whom they have weak ties (31). In addition, (32) reported that the most trusted sources for patients were specialist doctors, primary care providers, and nurses. Studies on the effects of trust have found that it leads to more positive attitudes (e.g., satisfaction) and behaviors (e.g., knowledge sharing) (33). Therefore, the effect of online health information on satisfaction is lower as compared to doctors' recommendations and family and friends' recommendations. Hence, we hypothesize that:

***H1****: The use of online health information sources has a negative impact on patient satisfaction*.

### Moderating effects

Previous studies have shown that patient-related characteristics (i.e., age, gender, education, socio-economic status, race, etc.) can affect patient satisfaction with health services (17, 34). In general, when patients need to make medical decisions, they will obtain health information from different sources (14, 22, 23). Online health information seeking is an increasingly popular way for patients to access information. Patients who use online health information sources are not limited by time or location, so they can quickly access health-related information to help them make decisions (1, 2). People seek health information, but if they find that one source (such as the Internet) cannot effectively meet their specific needs, they will turn to other sources (such as doctors). In evaluating the impact of various information sources on decision quality, we evaluated patient satisfaction after a visit to the doctor.

#### Doctor quality as a moderator

Doctor quality has been defined as the doctor's service meeting and/or exceeding a patient's expectations (35). According to the expectation–confirmation theory, when a doctor's service quality perceptions exceeds expectations, the patient will be satisfied with the doctor's service (25, 36). Many previous studies have shown that product/service quality is an important determinant of satisfaction (36, 37). In the healthcare context, this finding is also supported (16, 17, 38). Accordingly, the quality of a doctor's service has a positive impact on patient satisfaction. Therefore, the doctor quality directly affects patient satisfaction with treatment effects and service attitude. Hence, we hypothesize that:

***H2a****: Doctor quality has a positive impact on patient satisfaction*.

Many platforms provide product reviews, such as movie reviews on IMDB, restaurant reviews on Yelp, and teacher and perfessor reviews on Rate My Professors, which can be used as a basis for consumer decisions. In general, online product reviews are regarded as an effective proxy for quality and as having the potential to influence consumers' decision making (20, 39). In recent decades, many empirical studies have shown that online product reviews influence consumer decision-making and ultimately affect product sales (40–42). For example, in the film industry, online movie reviews have a significant positive effect on box office revenues (40–43). Zhu and Zhang (44) also show that online consumer reviews have a significant positive impact on product sales in the video game industry. Moreover, similar results have been found in the healthcare service context, where doctors' online reviews are one of the most important factors influencing patient behavior (45). In the OHC context, patients can find a lot of information about their disease and other patients' experience, as well as information about a doctor's online review on the OHC. If patients include the doctor's online review as a consideration in the decision-making process, it will improve the accuracy of their decision and thus increase their satisfaction. Therefore, the doctor quality (i.e., ratings of doctors) can reduce the negative effect between the use of online health information sources and patient satisfaction. Thus, we hypothesize that:

***H2b:*** *Doctor quality as a moderating effect will weaken the negative relationship between the use of online health information sources and patient satisfaction*.

#### Doctor popularity as a moderator

Doctor popularity is defined as the number of views of the doctor's webpages on the OHC (46). In general, doctor popularity is considered to be a signal of quality, i.e., the higher the popularity, the higher the quality of the doctor (44), implying that popularity may have a positive effect on satisfaction. Conversely, doctor popularity may lead to herding behavior, that is, regardless of individual needs or the doctor's service quality, patients will follow the crowd choice (47, 48). From a psychological point of view, such an unreasonable decision would be typical herding behavior. Herding behavior generates excessive expectations, leading to greater disconfirmation, and therefore plays a large role in negative outcomes (49, 50). Customers choose popular products by following the opinions of others rather than making a wellthought-out decision, and once they understand the product after using it, they are more likely to feel regretful afterwards. Therefore, herding behavior can lead to a decline in customer satisfaction. Previous research has supported the idea that popularity has a significant negative impact on satisfaction in the durable product context (51). Hence, we hypothesize that:

***H3a****: Doctor popularity has a negative impact on patient satisfaction*.

From the perspective of the herding theory, patients are usually influenced by the majority group and follow the thoughts or behaviors of the general public. As a result, patients may choose popular doctors that do not meet their needs or are not suitable for their situation at a certain time, leading to disappointment and dissatisfaction (52). As a consequence, herding behavior (i.e., choosing a popular doctor) can strengthen the negative effects of online information on satisfaction as compared to the choice of a doctor with less popularity. Therefore, patients who use online information to find a more popular doctor experience less satisfaction after their visit, as compared to a doctor with less popularity. This means that patients will have higher expectations due to their choice of a popular doctor, and therefore, if expectations exceed the perception of a doctor's quality of the service, patients will be less satisfied with the doctor. Thus, we hypothesize that:

***H3b:*** *Doctor popularity as a moderating effect will strengthen the negative relationship between the use of an online health information source and patient satisfaction*.

#### Patient involvement as a moderator

Patient involvement is related to the personal relevance or importance of medical decision-making (53). In the medical context, involvement means participation in decision-making, whether it involves personal care or is high-level decision-making (i.e., surgery). Many previous studies have explored how patient involvement can increase patient satisfaction (54–56). Similarity, involvement and satisfaction have been explored frequently in the consumer literature, and it is generally assumed that that higher levels of involvement are associated with higher levels of satisfaction (54, 57, 58). Hence, we hypothesize that:

***H4a****: Patient involvement has a positive impact on patient satisfaction*.

It is important to understand the level of patient participation in the care and treatment decision-making process; higher participation means higher involvement (59). Patients become more involved search for clues related to a specific disease in order to make appropriate decisions; because of the increased efficacy and the efficiency this process represents, it facilitates their medical decisions. In general, the greater risk involved in more difficult decision-making tasks influences the types of information sources that patients seek (60). As decision-making tasks become more difficult, patients are less confident in their ability to make good judgments, so they spend more time and effort seeking information that will further increase their confidence in their medical decisions. Thus, patient involvement will weaken the negative effect of the use of online health information sources on patient satisfaction. The negative relationship between using online health information sources and satisfaction is weakened because increased patient involvement can reduce the risk of uncertainty and increase the correctness of decisions. Hence, we hypothesize that:

***H4b:*** *Patient involvement as a moderating effect will strengthen the positive relationship between the use of an online health information source and patient satisfaction*.

## Research methods

### The research context

This study used the OHC as the research context. The primary reasons for this are as follows: first, the OHC has become an important doctor-patient interaction platform. It is used not only by patients to share doctor reviews and personal experience but also by doctors to provide professional medical information and consulting services (61–63). In addition, the OHC is a potential solution to the problem of the medical disparities that exist between urban and rural areas (64). Second, with the development of a mature Internet, the OHC has become more and more popular and is now a common way for patients to explore healthcare options online (14, 65). In recent years, a large amount of research data has been accumulated, and it has attracted the attention of many researchers (14, 16, 61, 64–67).

Haodf (Haodf means “good doctor” in Chinese) is the largest doctor-patient interaction platform in China and is considered to be the most professional and trustworthy of the OHCs. More than 490 thousand physicians from 7,500 hospitals nationwide are represented on the website; of these, 145 thousand physicians had completed real-name registration on the website in 2017. Therefore, Haodf is a very valuable reference website to help patients understand their needs and choose a doctor. More and more patients use this website to evaluate and choose doctors online.

The current study used the Haodf website (i.e., www.haodf.com) to test the hypotheses for two primary reasons: (1) the website provides a lot of data for the current research. It has many features (i.e., sources of information for choosing a doctor, information on the popularity of doctors) that make it particularly suitable for testing our proposed model, and the site has become popular among many doctors and patients; (2) the website provides a platform where doctors and patients can interact with each other. Patients can consult with their doctors and make offline appointments. Based on the results of their visits to the hospital, they can evaluate service attitude satisfaction and treatment effect satisfaction. Therefore, we can examine the factors that affect patient satisfaction.

### Data collection and processing

Our sample was collected from the Haodf website, which is one of the most wellknown online healthcare communities in China. This OHC lets patients share their treatment experiences with others by writing reviews of their doctors. On each review page, patients can rate their satisfaction with the treatment effects and the service attitude. The Haodf website also provides a standard option to answer the question “Why did you choose this doctor,” which allowed us to learn what information source patients had used to choose that doctor. Besides the reviews, some of the characteristics or behaviors of the doctors in OHC are also worth noting. Specifically, doctors play an important role in OHC, not only by writing articles to share certain medical knowledge but also by providing online healthcare services to patients. In order to ensure the accuracy of the doctor's identity, every doctor must be certified by the Haodf website. As of December 2019, more than 230,000 doctors have completed their registration on this platform. Since the basic information regarding these doctors and the records of their behavior on the website are public data, we can combine the patient review data with the doctor-related data for analysis. Details regarding data collection and processing are shown below.

**Figure 2:**
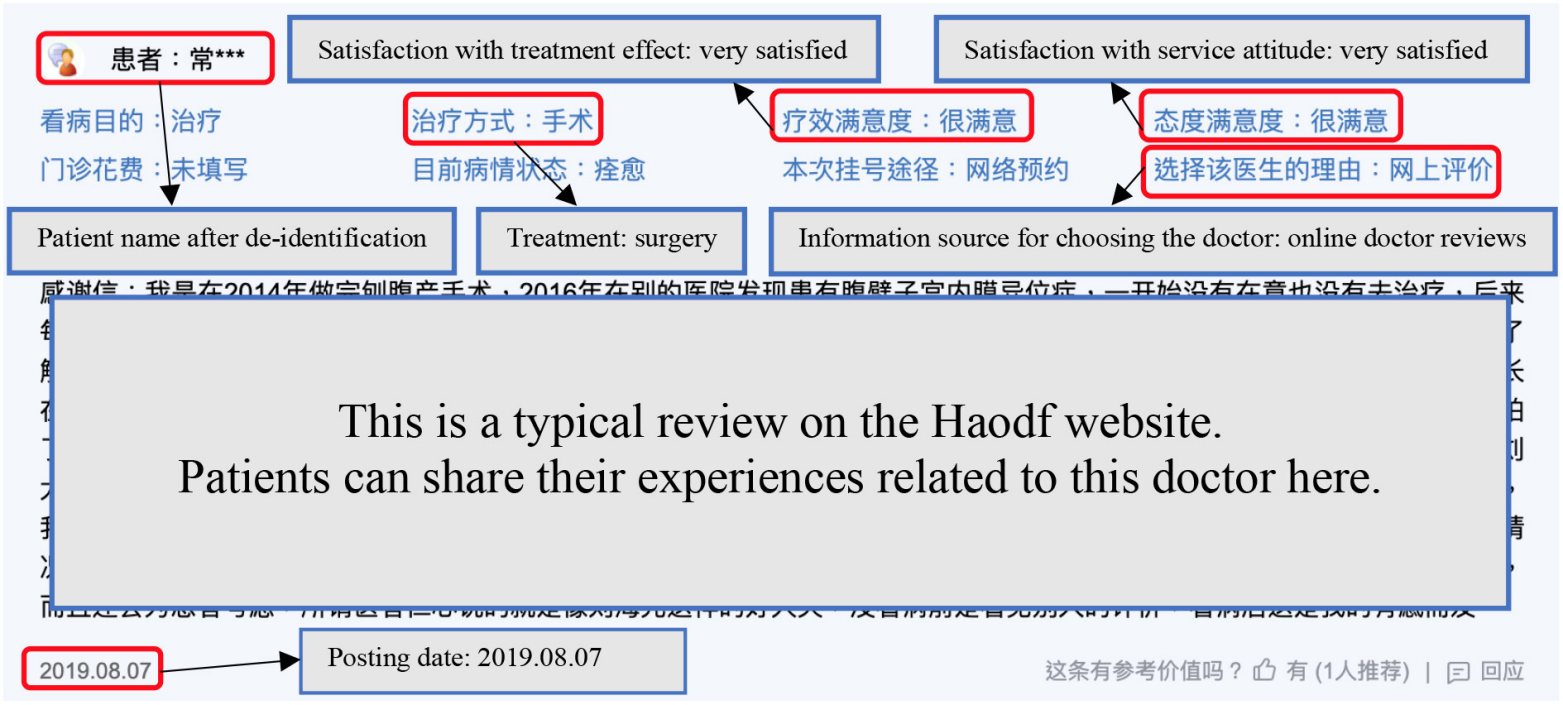
An example of an online doctor review.

The process of data collection was divided into two parts. First, in August 2017, we started recording information on ~140,000 doctors from the Haodf website; this included information such as clinic title, hospital affiliation, online rating scores, and the number of the doctor's home page views. The same process was repeated once a month until July 2019. In other words, we collected each doctor's public profile and OHC statistics on a monthly basis over a two-year period. We then collected data on all reviews from September 1, 2017, to August 31, 2019. Figure 2 represents an example of a typical online doctor review, with each review containing textual content, a de-identified patient name, the posting date, the treatment approach, the information source used for choosing that doctor, satisfaction with the treatment effect, and satisfaction with the service attitude. Depending on which doctor each review corresponded to and when it was posted, we attempted to merge this review with the previous month's data for the corresponding doctor. After removing incomplete data, the final sample that remained was 952,877 online doctor reviews, evaluating satisfaction with 54,027 doctors from 3,525 hospitals in 31 provinces.

### Measures

#### The dependent variable

##### Patient satisfaction

The dependent variable in this study was patient satisfaction. There are two types of patient satisfaction, namely, treatment effect satisfaction (*PS*_1_) and service attitude satisfaction (*PS*_2_) (16). We used online doctor reviews given by patients to indicate patient satisfaction. We define *PS*_1_ and *PS*_2_ as dummy variables, in which the patient's evaluation of the doctor as “very satisfied” was coded as 1 and other evaluations were coded as 0.

#### The independent variable

##### Information sources

In this study, there were three different information sources for patients to choose a doctor: online reviews, family/friend recommendations, and doctor recommendations (14). With the proliferation of the Internet, online health information sources are becoming increasingly important. Therefore, we wanted to further understand the interaction effect of online health information source on patient satisfaction. We used a dummy variable for online health information source, coded as 1 if the source of health information was from the Internet and 0 otherwise.

#### Moderator variables

##### Doctor quality

The quality of the doctor was measured using the star score listed on the Haodf website (20, 39, 61). *DQ* is defined as a dummy variable, with a doctor's star score greater than the median of all doctors' star score coded as 1, and all other coded as 0.

##### Doctor popularity

The popularity of the doctor was measured by the number of page views (46). *DP* is defined as a dummy variable, where a doctor's page view number greater than the median number of page views for all doctors was coded as 1, and other numbers were coded as 0.

##### Patient involvement

The involvement of the patient was measured by whether the patient needed surgery. In general, the level of involvement in treatment requiring surgery is higher than that in treatment not requiring surgery (68). *INV* is defined as a dummy variable, in which a patient's treatment requiring surgery was coded as 1, and that not requiring surgery was coded as 0.

#### Control variables

We employed five control variables in this study. First, the doctor's offline reputation was measured using the doctor's clinic title, which indicates the medical ranking of the doctor as it is evaluated by the government according to his or her comprehensive abilities. In China, the doctor's clinic title (*CL*) can be specified as chief physician, associate chief physician, attending physician, or resident physician. We defined *CL* as a dummy variable, in which the position of chief physician was coded as 1 and others were coded as 0 (14). Second, the hospital level (*HL*) was based on the official certification of the hospital, which reflects a hospital's equipment, functions, and technology, etc. In this study, *HL* is a dummy variable that was set to 1 if the doctor was from a tertiary hospital and 0 otherwise (62). Third, the city level (*CITY*) reflects the consumption level of consumers, which may affect consumer satisfaction. *CITY* is also a dummy variable, taking the value 1 if the doctor was from a resource-rich city (i.e., Beijing, Shanghai, Shenzhen, or Guangdong) and 0 otherwise (14). Fourth, as the Haodf website has been established for more than 10 years, the length of time a doctor (*RT*) has been a member on the site may influence his or her behavior on it. To control for time effects, *RT* was defined as the doctor's relationship time with the Haodf website, which was measured by the number of days since the doctor had first joined the website (66). Finally, doctors have different major specialty areas (*DIV*) such as internal medicine, surgery, pediatrics, and traditional Chinese medicine. To reduce the influence of a doctor's specialty characteristics, we added another control variable, *DIV*, which is a categorical variable with 10 major divisions, as reported on the website (62). Table 1 shows the detailed variable definitions and their measurements.

**Table 1:** Variable definitions and measurements.

**Variables**	**Definitions**	**Measurements**
**Dependent variables**		
PS1_t_	Treatment effect satisfaction	A dummy variable, coded 1 if the patient rated the doctor's treatment effect as “very satisfied,” and 0 otherwise.
PS2_t_	Service attitude satisfaction	A dummy variable, coded 1 if the patient rated the doctor's service attitude as “very satisfied,” and 0 otherwise.
**Independent variables**		
IS_t−1_	Information source	A dummy variable, coded 1 if the source of health information is from online, and 0 otherwise.
**Moderator variable**		
DQ_t−1_	Doctor quality	A dummy variable, coded 1 if the doctor's star score is greater than the median of star score for all doctors, and 0 otherwise.
DP_t−1_	Doctor popularity	A dummy variable, coded 1 if the doctor's page views are greater than the median number of page views for all doctors, and 0 otherwise.
INV_t−1_	Patient involvement	A dummy variable, coded 1 if the patient's treatment requires surgery, and 0 otherwise.
**Control variables**		
CL_t−1_	Clinic title	A dummy variable, coded 1 if the doctor's clinic title is chief physician or associate chief physician, and 0 otherwise.
HL_t−1_	Hospital level	A dummy variable, coded 1 if the doctor is from a tertiary hospital, and 0 otherwise.
CITY_t−1_	City level	A dummy variable, coded 1 if the doctor comes from Beijing or Shanghai or Guangdong or Shandong, and 0 otherwise.
RT_t−1_	Relationship time with Haodf	The doctor's tenure with the Haodf website (in days), calculated by data download date minus this doctor's registration date on the website.
DIV_t−1_	Division	The doctor's division as categorized by the website, including internal medicine, surgery, pediatrics, traditional Chinese medicine, orthopedics, gynecology-obstetrics, oral health, ophthalmology, cancer, and others.

#### Statistical analysis: logistical regression analysis

To test the proposition of this paper, we express our base model as follows.


(1) $$PS_{i,g}\;=\;\alpha_{g}\;+\;\beta_{g}IS_{i,g}\;+\;\sum_{j=1}^{J}\gamma_{g}^{j}MV_{i,g}^{j}\;+\;\sum_{k=1}^{K}\delta_{g}^{k}CV_{i,g}^{k}\;+\;\varepsilon_{g}$$

*where* $i=1,2,\cdots \;,N;\;j=1,2,\cdots \;,J;\;k=1,2,\cdots \;,K;\;\varepsilon_{g\;}\sim iid\;N\left(0,\sigma_{g}^{2}\right);\;g=1\;or\;2$.

In Equation (1), *PS*_i, g_ on the left of the equal sign is patient satisfaction, the subscript *i* is the *i*-th review, and the subscript g represents the type of patient satisfaction; where *g* may 1 or 2, representing treatment effect satisfaction (*PS*_1_) and service attitude satisfaction (*PS*_2_), respectively. Next, α denotes the intercept, β denotes the coefficient of information resources (*IS*), and γ and δ are vectors of the parameters to be estimated. Furthermore, *IS* is an independent variable, *MV* is a vector of multiple main variables as a set of moderator variables, and *CV* is a vector of multiple doctor characteristics as a set of control variables. The distribution term ε follows a normal distribution, which makes the regression a logistical regression. The dependent variables are collected at time *t*, and moderator variables as well as all independent and control variables are calculated using the data collected at time *t*-1.

To investigate the moderating effect of *IS*, we further considered a logistical regression model with interaction terms, shown as Model (2).


(2) $$\begin{array}{l} PS_{i,g}\;=\;\alpha_{g}\;+\;\beta_{g}IS_{i,g}\;+\;\sum_{j\;=\;1}^{J}\gamma_{g}^{j}MV_{i,g}^{j}\;+\;\sum_{k=1}^{K}\delta_{g}^{k}CV_{i,g}^{k}\\ \;+\;\zeta_{g}^{l}IS_{i,g}\sum_{l=1}^{L}MV_{i,g}^{l}+\varepsilon_{g} \end{array}$$

$where\;i=1,2,\cdots \;,N;\;j=1,2,\cdots \;,J;\;k=1,2,\cdots \;,K;\;l=1,2,\cdots \;,L;\;\varepsilon_{g\;}\sim iid\;N\left(0,\sigma_{g}^{2}\right);$

## Results

### Descriptive statistics and preliminary findings

As shown in Table 2, the descriptive statistics of our major variables include doctors' personal information, information sources, and patients' satisfaction with treatment effects and service attitude. Table 3a shows the odds ratio (*OR*) of treatment effect satisfaction for each factor. The *OR* of patients using online information sources' being very satisfied with the treatment effect can be calculated as (291,466 × 38,316)/(600,119 × 22,976) = 0.81, meaning that the number of patients using online health information sources indicating they were very satisfied with the treatment effect was 0.81 times lower than that of patients using other information sources. Similarly, we can calculate *OR* as (365,349 × 36,062)/(526,236 × 25,230) = 0.99, which means that the chance of a high-quality doctor making patients very satisfied with the treatment effect was approximately equal to that of a low-quality doctor. In terms of doctor popularity, the OR was (443,651 × 28,506)/(447,934 × 32,786) = 0.86, meaning that a high-popularity doctor was 0.86 times less likely to make patients very satisfied with the treatment effect than a low-popularity doctor. In addition, we also calculate *OR* as (438,874 × 45,280)/ (452,711 × 16,012) = 2.74, meaning that patients with high-involvement treatment were 2.74 times more likely to have a very satisfactory treatment effect than those with low-involvement treatment. Table 3b shows the *OR* of service attitude satisfaction for each factor. The *OR* of patients using online health information sources being very satisfied with the service attitude can be calculated as (302,231 × 21,806)/(616,629 × 12,211) = 0.88, meaning that the number of patients using online health information sources who indicated they were very satisfied with their service attitude was 0.88 times lower than that of patients using other information sources. Similarly, we can calculate *OR* as (377,195 × 20,633)/ (541,665 × 13,384) = 1.07, which means that the chance of a high-quality doctor making patients very satisfied with the service attitude was approximately equal to that of a low-quality doctor. In terms of doctor popularity, the OR was (443,651 × 28,506)/(447,934 × 32,786) = 0.85, meaning that a high-popularity doctor was 0.85 times less likely to make patients very satisfied with the service attitude than a low-popularity doctor. In addition, we also calculate *OR* as (438,874 × 45,280)/(452,711 × 16,012) = 1.77, meaning that patients with high-involvement treatments were 1.77 times more likely to be very satisfied with their service attitude than those with low-involvement treatments.

**Table 2:** Descriptive statistics of variables.

**Variable**	**Mean**	**Std. Dev**.	**Minimum**	**Maximum**
Treatment effect satisfaction (PS_1t_)	0.936	0.245	0	1
Service attitude satisfaction (PS_2t_)	0.964	0.186	0	1
Information source (IS_t−1_)	0.330	0.470	0	1
Doctor quality (DQ_t−1_)	0.410	0.492	0	1
Doctor popularity (DP_t−1_)	0.500	0.500	0	1
Patient involvement (INV_t−1_)	0.477	0.499	0	1
Clinic title (CL_t−1_)	0.352	0.478	0	1
Hospital level (HL_t−1_)	0.928	0.259	0	1
City level (CITY_t−1_)	0.464	0.500	0	1
Relationship time with Haodf (RT_t−1_)	2,027.340	1,076.424	9	4,197

The sample size is 952,877 online doctor reviews, which evaluate the satisfaction for 54,027 doctors from 3,525 hospitals or 31 provinces.

**Table 3:** Summary of the odds ratio of patient satisfaction for main factors.

**Variables**		**Treatment effect satisfaction**		**Total**
		**1 (very satisfied)**	**0 (others)**	
**a. Treatment effect satisfaction**				
Information sources	1 (online)	291,466	22,976	314,442
	0 (others)	600,119	38,316	638,435
Doctor quality	1 (high)	365,349	25,230	390,579
	0 (low)	526,236	36,060	562,298
Doctor popularity	1 (high)	443,651	32,768	476,437
	0 (low)	447,934	28,506	476,440
Patient involvement	1 (high)	438,874	16,012	454,886
	0 (low)	452,711	45,280	497,991
Total	891,585	61,292	952,877
**b. Service attitude satisfaction**				
**Variables**		**Service attitude satisfaction**		**Total**
		**1 (very satisfied)**	**0 (others)**	
Information sources	1 (online)	302,231	12,211	314,442
	0 (others)	616,629	21,806	638,435
Doctor quality	1 (high)	377,195	13,384	390,579
	0 (low)	541,665	20,633	562,298
Doctor popularity	1 (high)	458,123	18,314	476,437
	0 (low)	460,737	15,703	476,440
Patient involvement	1 (high)	443,143	11,743	454,886
	0 (low)	475,717	22,274	497,991
Total	918,860	34,017	952,877

### Logistical regression analysis results: treatment effect satisfaction

Table 4 summarizes the results of our model estimated by binomial logistic regression. The model was estimated hierarchically; we first introduced the model with independent and control variables, and then we tested the full model with the interaction terms in column 2. As shown in column 1 of Table 4, we found that the information source had negative and significant effects on patient satisfaction with treatment effect (β = −0.173, *p* < 0.001). Therefore, H1 was supported. This indicates that patients' satisfaction with treatment effect was lower when they used online health information sources to select a doctor. Similarly, patients' satisfaction with treatment effect was higher when they used other information sources (e.g., recommendations from family and friends or physician recommendations). Thus, online health information sources had a negative effect on treatment effect satisfaction. The results showed that the doctor quality had a positive effect on treatment effect satisfaction (β = 0.082, *p* < 0.001 in Model 1). Thus, H2a was supported. The effect of the doctor popularity on treatment effect satisfaction was negative and significant; thus, H3a was supported (β = −0.125, *p* < 0.001 in Model 1). Regarding decision involvement, there was a positive effect on treatment effect satisfaction (β = 0.907, *p* < 0.001 in Model 1); thus, H4a was supported.

**Table 4:** Results for the effects of determinants on treatment effect satisfaction.

**Independent variables**	**Model 1**	**Model 2**
Intercept	2.318^***^ (0.052)	2.304^***^ (0.052)
**Main effects**		
Information source (IS)	−0.173^***^ (0.009)	−0.111^***^ (0.015)
Doctor quality (DQ)	0.082^***^ (0.010)	0.101^***^ (0.012)
Doctor popularity (DP)	−0.125^***^ (0.011)	−0.069^***^ (0.013)
Patient involvement (INV)	0.907^***^ (0.010)	0.836^***^ (0.012)
**Interaction effects**		
IS ^*^ DQ		−0.046^*^ (0.019)
IS ^*^ DP		−0.164^***^ (0.020)
IS ^*^ INV		0.204^***^ (0.020)
**Control variables**		
Clinic title (CL)	−0.140^***^ (0.009)	−0.141^***^ (0.009)
Hospital level (HL)	−0.209^***^ (0.017)	−0.209^***^ (0.017)
City level (CITY)	0.074^***^ (0.009)	0.076^***^ (0.009)
Relationship time (RT)	0.048^***^ (0.007)	0.048^***^ (0.007)
Surgery	0.129^***^ (0.015)	0.131^***^ (0.015)
Pediatrics	0.150^***^ (0.017)	0.149^***^ (0.017)
Traditional Chinese medicine	−0.238^***^ (0.018)	−0.236^***^ (0.018)
Orthopedics	−0.160^***^ (0.020)	−0.158^***^ (0.020)
Gynecology-obstetrics	0.089^***^ (0.020)	0.087^***^ (0.020)
Oral health	0.285^***^ (0.030)	0.282^***^ (0.030)
Ophthalmology	−0.140^***^ (0.023)	−0.143^***^ (0.023)
Cancer	0.320^***^ (0.030)	0.322^***^ (0.030)
Others	−0.322^***^ (0.014)	−0.320^***^ (0.014)

* p < 0.05,

^**^p < 0.01,

*** p < 0.001.

Standard error in parentheses.

The moderating effects of the doctor quality on the effect of information source on treatment effect satisfaction was negative (β = −0.046, *p* < 0.05 in column 2 of Table 4), not supporting H2b. The moderating effects of the doctor popularity on the effects of information source on treatment effect satisfaction was negative (β = −0.164, *p* < 0.001 in Model 2), while conversely, the moderating effects of patient involvement on the effect of information source on treatment effect satisfaction was positive (β = 0.204, *p* < 0.001 in Model 2); thus, H3b and H4b were supported.

**Figure 3:**
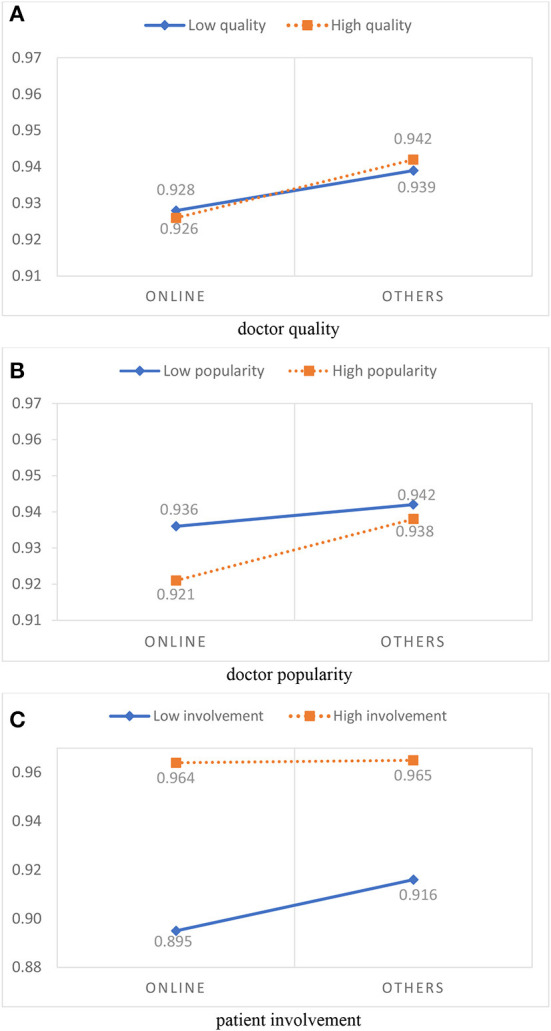
Moderation effects of online health information sources on treatment effect satisfaction. **(A)** Doctor quality. **(B)** Doctor popularity. **(C)** Patient involvement.

In addition, to illustrate the interaction effects more clearly, the interaction diagram is shown in Figure 3. Figure 3A shows that the effects of information source on treatment effect satisfaction (*PS*_1_) were found to be negative under both the low-quality doctor condition and high-quality doctor condition. The growth rate of *PS*_1_ of high-quality doctors (the dotted line) is slightly higher than that for low-quality doctors (the solid line), indicating that doctor quality moderates the correlation between online health information sources and treatment effect satisfaction (*PS*_1_). As shown in Figure 3B, when online information was used to select high-popularity doctors (the dotted line), *PS*_1_ was significantly lower than it was doctors with low- popularity (the solid line), indicating that high popularity of the doctor strengthened the negative effect of using an online information source on treatment effect satisfaction (*PS*_1_). As shown in Figure 3C, for patients receiving high-involvement treatments (the dotted line), *PS*_1_ was higher than it was for patients receiving low-involvement treatments (the solid line), regardless of the use of online health information sources or other information sources, indicating that patient involvement weakens the negative effect of using an online information source on treatment effect satisfaction (*PS*_1_).

### Logistical regression analysis results: service attitude satisfaction

The results for service attitude satisfaction were similar, except that the moderating effect of the information source on the effect of doctor quality on service attitude satisfaction was not significant, as shown in Table 5. As shown in column 1 of Table 5, we found that the information source had negative and significant effects on service attitude satisfaction (β = −0.102, *p* < 0.001). This indicates that service attitude satisfaction was lower when they used online health information sources to select a doctor. Thus, online health information sources have a negative effect on service attitude satisfaction. The results showed that the doctor quality had a positive effect on service attitude satisfaction (β = 0.179, *p* < 0.001). The doctor popularity had a negative and significant effect on service attitude satisfaction (β = −0.123, *p* < 0.001); conversely, patient involvement had a positive effect on service attitude satisfaction (β = 0.528, *p* < 0.001). The moderating effects of information source on the effects of the doctor popularity on service attitude satisfaction was negative (β = −0.198, *p* < 0.001), while conversely, the moderating effects of information source on the effects of decision involvement on service attitude satisfaction was positive (β = 0.111, *p* < 0.001).

**Table 5:** Results for the effects of determinants on service attitude satisfaction.

**Independent variables**	**Model 1**	**Model 2**
Intercept	3.502^***^ (0.070)	3.482^***^ (0.071)
**Main effects**		
Information source (IS)	−0.102^***^ (0.012)	−0.013 (0.021)
Doctor quality (DQ)	0.179^***^ (0.013)	0.190^***^ (0.016)
Doctor popularity (DP)	−0.123^***^ (0.014)	−0.057^***^ (0.017)
Patient involvement (INV)	0.528^***^ (0.013)	0.490^***^ (0.015)
**Interaction effects**		
IS ^*^ DQ		−0.023 (0.026)
IS ^*^ DP		−0.198^***^ (0.026)
IS ^*^ INV		0.111^***^ (0.025)
**Control variables**		
Clinic title (CL)	−0.320^***^ (0.012)	−0.322^***^ (0.012)
Hospital level (HL)	−0.218^***^ (0.023)	−0.218^***^ (0.023)
City level (CITY)	0.050^***^ (0.012)	0.052^***^ (0.012)
Relationship time (RT)	0.005 (0.010)	0.005 (0.010)
Surgery	−0.071^***^ (0.020)	−0.070^***^ (0.020)
Pediatrics	−0.043 (0.023)	−0.045^*^ (0.023)
Traditional Chinese medicine	−0.185^***^ (0.026)	−0.183^***^ (0.026)
Orthopedics	−0.213^***^ (0.027)	−0.211^***^ (0.027)
Gynecology-obstetrics	−0.174^***^ (0.026)	−0.175^***^ (0.026)
Oral health	−0.074^*^ (0.036)	−0.075^*^ (0.036)
Ophthalmology	−0.202^***^ (0.030)	−0.204^***^ (0.030)
Cancer	0.196^***^ (0.040)	0.199^***^ (0.040)
Others	−0.312^***^ (0.020)	−0.310^***^ (0.020)

* p < 0.05,

^**^ p < 0.01,

*** p < 0.001. Standard error in parentheses.

**Figure 4:**
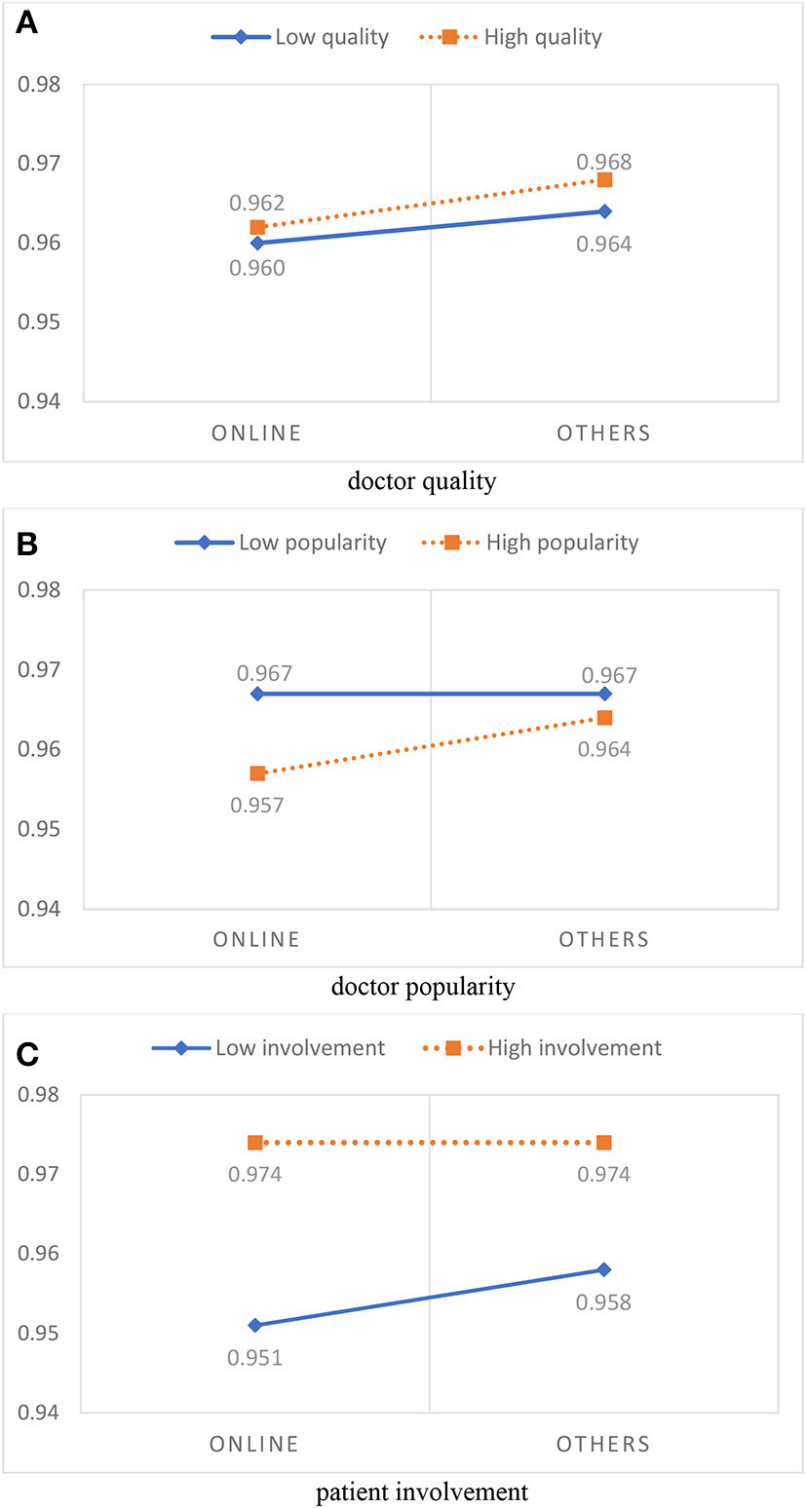
Moderation effects of online health information sources on service attitude satisfaction. **(A)** Doctor quality. **(B)** Doctor popularity. **(C)** Patient involvement.

The interaction diagram is shown in Figure 4. Figure 4A shows that the growth rate of the *PS*_1_ of high-quality doctors (the dotted line) was almost the same as that of low-quality doctors (the solid line), indicating that doctor quality does not moderate the correlation between online health information sources and service attitude satisfaction (*PS*_2_). Figure 4B shows that the effects of the doctor popularity on service attitude satisfaction was found to be significant under both the online information source condition and the other information source condition, but *PS*_2_ was clearly lower when high-popularity doctors (the dotted line) were selected than when low-popularity doctors (the solid line) were selected using online information, which indicates that a high-popularity doctor strengthened the negative effect of using an online information source on service attitude satisfaction (*PS*_2_). As shown in Figure 4C, regardless of the use of online health information sources or other information sources, the *PS*_2_ of patients with high-involvement treatments (the dotted line) was higher than that of patients with low-involvement treatments (the solid line), indicating that patient involvement weakened the negative effect of online information source on service attitude satisfaction (*PS*_2_).

## Discussion and implications

### Theoretical implications

In this study, we examined the effect of using different information sources on patient satisfaction. We found that using online health information sources to find a doctor resulted in lower patient satisfaction (both with the treatment effect and with service attitude) after the patient had seen a doctor than other information sources. In addition, we found that doctor popularity and patient involvement moderated the relationship between information source and patient satisfaction. However, the moderating effect of doctor quality was mixed. This study makes several methodological and theoretical contributions. First, in terms of methodology, previous studies on patient satisfaction have used cross-sectional questionnaires and descriptive studies (69, 70), which may be geographically limited and make it difficult to establish a causal relationship between determinants and satisfaction. Batbaatar et al. (17) and Rains (18) indicated that a longitudinal study design is needed to detect causal relationships. Thus, this study used longitudinal data, collected monthly for two consecutive years, and included 54,027 doctors and 952,877 online doctor reviews from 3,525 hospitals in 31 provinces. The comprehensive results can be better understood as indicating cause-and-effect relationships affecting patient satisfaction.

Second, many studies related to recommendation sources [e.g., (19, 20)] have shown that online consumer reviews are the main source of information for consumers' decisions. Previous studies have focused on the impact of online consumer reviews on consumer choice, and few studies have examined customers' post-purchase satisfaction when they use different information sources. To the best of our knowledge, this study is one of the first to use a large amount of OHC data to explore the impact of information sources on patient satisfaction. We found the interesting result that patient satisfaction was generally lower among patients who found their doctors through online health information sources.

Third, we found that the moderating effect of doctor quality was mixed, a finding that did not support our hypothesis. One of the possible reasons for this is that finding the right doctor through the Internet allows patients to quickly and more comprehensively understand the quality of the doctor, thus positively affecting patient satisfaction (39). On the other hand, according to expectation-confirmation theory (36), patients have higher expectations of doctors after learning about their quality, which increases their disconfirmation and leads to negative outcomes for patient satisfaction. In addition, doctor popularity and patient involvement have significant moderating effects on the relationship between information sources and patient satisfaction. Doctor popularity may be determined by people's herding choices, known as herding behavior, without regard to individual needs or the quality of the physician (47, 48). According to expectation-confirmation theory, herding behavior creates excessive expectations that lead to greater disconfirmation, thus reinforcing the negative impact of online health information sources on patient satisfaction (49). Thus, patients who use online information to find a more popular doctor have lower satisfaction after their visit, compared to those who find a less popular doctor. Conversely, patient involvement weakens the negative impact of online health information sources on patient satisfaction. In general, the increased risk involved in more difficult decision-making tasks affects the type of information sources that patients seek (60). Patients become more involved in finding disease-specific clues to make appropriate decisions; their medical decisions are facilitated by the increased efficacy and efficiency represented by this process. Thus, the negative relationship between the use of online health information sources and patient satisfaction is diminished because increased patient involvement reduces the risk of uncertainty and increases satisfaction with decision making. Our empirical evidence supports the significance of both moderator variables. Therefore, future studies of the effects of patient information seeking on satisfaction should consider adding both doctor popularity and patient involvement to their research models.

### Managerial implications

Based on our results, we have provided some management implications for policies, OHC management, and patients. First, the Internet has become an important channel through which users can obtain health information (14, 15). Seeking, understanding, and using health information is essential for health decision-making. People who want to solve medical problems will seek health information to understand the risk factors, and they will learn preventive measures (15, 71). However, the results of our research indicated that the satisfaction resulting from choosing a doctor through online information is low. A possible reason is that the information on the Internet may be wrong or inaccurate. Therefore, relevant professional departments should monitor and evaluate the quality of information and enhance the accuracy of online health information.

Second, many patients may select a more highly-popular doctor due to their quality or due to mindless herd behavior. Our results show that doctor popularity has a negative moderating effect on information sources and patient satisfaction. In other words, the satisfaction level from popular doctors is significantly lower than that from non-popular doctors for patients who seek doctors, especially through online information. Thus, patients should attempt to break the “bandwagon effect” and choose a doctor that meets their needs. In addition, from the perspective of the OHC manager, the recommendation system can be used to increase the exposure of low-popular doctors, so that patients can also filter by region, gender, specialty, etc., and find doctors that are more suitable for them.

Third, patient involvement is a priority for healthcare information seeking to improve the quality of care and services. The results of this study show that patient participation has a positive moderating effect on information sources and patient satisfaction. Many past studies have examined the benefits of patient involvement, such as increased patient satisfaction and trust, improved quality of life, better understanding of individual needs, and reduced patient anxiety and mood (55, 56, 59). Therefore, we should provide a more friendly communication environment, encourage patient participation, enhance trust in services, improve information transparency, and thus reduce the likelihood of patient dissatisfaction (59, 72).

### Limitations and directions for future research

There are some limitations to this study and possible directions for future studies. First, due to the de-identification of patients by the OHC to protect their privacy, we were unable to closely track the progress of each patient. As a result, we were unable to learn whether patients had consulted multiple doctors at the same time. Future studies could collect more detailed data to compare the attitudes and quality of treatment of different doctors. Second, we included only patients' ratings of doctor satisfaction and did not analyze unstructured textual content. It is worth exploring the possibility that sentiment analysis using a text mining procedure or natural language processing techniques might produce richer information about doctor-patient interaction and suggest more effective management strategies (16, 73). Third, all empirical data were collected from the Haodf website. Although the website is representative of China, this means that our findings may reflect only the impact of China's online information seeking on patient satisfaction in the healthcare context. Past studies have pointed out that cultural factors in different countries may be important factors influencing consumer rating behavior (43, 74). Future studies may include different countries to increase the generalizability of their findings. Finally, some invisible syndromes may not be detectable through online health information, but it can be just as damaging to people's physical health or mental health (75). Therefore, it is also worthwhile to explore how the invisible syndrome can provide useful information through the Internet.

## Data availability statement

Publicly available datasets were analyzed in this study. This data can be found here: www.haodf.com.

## Author contributions

YS: investigation, resources, methodology, and writing—original draft preparation. JY: visualization, formal analysis, investigation, and conceptualization. Y-LC: writing—reviewing and editing and conceptualization. Y-TH: data curation and writing—original draft preparation. All authors contributed to the article and approved the submitted version.

## Funding

JY gratefully acknowledges the support by Guangzhou Huashang College Research Funds (2021HSKT04).

## Conflict of interest

The authors declare that the research was conducted in the absence of any commercial or financial relationships that could be construed as a potential conflict of interest.

## Publisher's note

All claims expressed in this article are solely those of the authors and do not necessarily represent those of their affiliated organizations, or those of the publisher, the editors and the reviewers. Any product that may be evaluated in this article, or claim that may be made by its manufacturer, is not guaranteed or endorsed by the publisher.

## Footnotes

^1^DXY is the abbreviation of Ding Xiang Yuan, formerly known as “Ding Xiang Yuan Medical Literature Retrieval Network,” which was established on July 23, 2000, as a medical knowledge sharing website (i.e., www.dxy.cn).
